# Good Practices and Challenges in the Collaboration of Pharmacists with General Practitioners—A Scoping Review

**DOI:** 10.3390/pharmacy14010024

**Published:** 2026-02-03

**Authors:** Evelina Gavazova, Kiril Atliev, Daniela Kafalova

**Affiliations:** 1 Department of Organisation and Economics of Pharmacy, Faculty of Pharmacy, Medical University of Plovdiv, 4000 Plovdiv, Bulgaria; daniela.kafalova@mu-plovdiv.bg; 2 Department of Epidemiology and Disaster Medicine, Faculty of Public Health, Medical University of Plovdiv, 4000 Plovdiv, Bulgaria; kiril.atliev@mu-plovdiv.bg

**Keywords:** primary care, pharmacists, general practitioners, collaboration, patient-centered care, scoping review

## Abstract

Optimizing medication management and improving patient health outcomes depend primarily on the strength of primary healthcare services, where collaboration between general practitioners (GPs) and pharmacists plays a critical role. This scoping review aimed to identify the main facilitators and barriers influencing pharmacist–GP collaboration. The review was conducted in line with PRISMA-ScR guidelines. A comprehensive search of PubMed, Scopus, and Web of Science identified studies published in English between January 2019 and May 2025, of which twenty met the inclusion criteria. Key facilitators of collaboration included pharmacist co-location within GP practices, clearly defined professional responsibilities, access to shared electronic health records, and supportive government policies. Barriers most frequently reported were limited communication pathways, insufficient interprofessional training, and financial constraints. Overall, the findings suggest that effective pharmacist–GP collaboration relies on structural integration, professional trust, and policy initiatives that enable sustained cooperation. Long-term investment in collaborative infrastructure and workforce development will be essential to strengthen primary care, support patient outcomes, and ensure more efficient use of healthcare resources.

## 1. Introduction

The patient’s first contact with healthcare is usually in primary care, as these settings play a crucial role in subsequent steps of the treatment process [1]. Primary care services shall ensure accessible, continuous, and comprehensive care, including prevention, diagnosis, treatment, and long-term management of medical conditions [2]. Sufficient and timely prevention and intervention through primary care can support patients‘ quality of life and decrease healthcare expenditures [3]. On the other hand, primary care contributes to population health education by promoting healthy lifestyles [4].

Today’s complex healthcare landscape poses challenges for general practitioners. The increased prevalence of chronic diseases and the aging population has led to high demand for services and increased workload for GPs [5]. Continuous professional development is required for GPs to remain familiar with up-to-date innovations and rapidly evolving technologies and developments in medicine [6]. The high burden is even more apparent in regions with a shortage of primary care professionals. Part of the time that should be spent with patients is devoted to administrative and insurance paperwork [6,7].

Without adequate support or resources, GPs are challenged to address complex mental and social health issues [8]. These challenges highlight the need for systemic support, better resource allocation, and investment in primary care infrastructure to ensure that general practitioners can continue to provide high-quality, patient-centered care [9]. One of the key tools for improving primary care, particularly for elderly and multimorbid patients, is the involvement of pharmacists in the care process [10]. Enhanced medication safety reduced inappropriate prescribing, and improved management of chronic diseases have been reported as major outcomes of integrating pharmacists into primary care [10,11,12]. Despite growing evidence supporting these collaborations, their implementation remains hindered by both structural and interpersonal challenges [13]. Experienced pharmacists specializing in pharmacotherapy are well-positioned to assist general practitioners in managing polypharmacy, chronic conditions, and medication-related problems [14]. Collaborative models involving pharmacists and GPs have shown potential to support medication safety, enhance patient outcomes, and optimize the use of healthcare resources [15]. Despite growing interest and supportive evidence, the implementation of pharmacist–GP collaboration remains uneven across different health systems [16,17,18]. While some countries have developed and funded models of integration, others face systemic, cultural, and logistical barriers. Successful collaboration requires not only structural support but also role clarity, mutual trust, shared goals, and effective communication between professionals.

This study aimed to explore and synthesize the good practices and challenges associated with pharmacist–GP collaboration, as reported in the recent empirical literature. By identifying common enablers and barriers, this review sought to inform future policy development, implementation strategies, and interprofessional training programs that can foster collaboration in primary care settings.

Given the wide variety of study designs, healthcare contexts, and collaborative models documented in the literature, a scoping review was identified as the most appropriate methodology for this investigation. The review’s objectives were exploratory: we aimed to chart the existing evidence, highlight best-practice examples and implementation challenges, and examine how pharmacist–general practitioner collaboration has been conceptualized and operationalized across diverse primary-care settings. The review addresses the following research questions. What good practices have been reported in pharmacist–GP collaboration in primary care? What challenges and barriers prevent efficient collaboration? What organizational factors influence the implementation of collaborative models?

## 2. Materials and Methods

The review follows the Preferred Reporting Items for Systematic Reviews and Meta-Analyses Extension for Scoping Reviews (PRISMA-ScR) guidelines.

### 2.1. Search Strategy

A literature search was conducted in PubMed, Scopus, and Web of Science to identify relevant studies examining collaboration between pharmacists and general practitioners. The search covered articles published between 1 January 2019 and 1 May 2025 and was limited to English-language publications.

The following search string was applied, with database-specific adaptations to syntax and indexing terms:

(“pharmacists” OR “clinical pharmacists”) AND (“general practitioners” OR “family physicians”) AND (“collaboration” OR “integration” OR “teamwork”)

Filters were applied where available to restrict results by publication date and language. Eligible publication types included original research articles, qualitative studies, mixed-methods studies, reviews, and implementation reports.

### 2.2. Eligibility Criteria

We used the following inclusion criteria: articles discussing collaboration between pharmacists and GPs in primary care or general practice settings; studies identifying either best practices (successful strategies and facilitators) or challenges/barriers to cooperation; peer-reviewed original research, reviews, or policy evaluations; and studies involving adult populations. The exclusion criteria were studies focused solely on hospital pharmacy or inpatient settings; editorials; opinion pieces; conference abstracts without full text; articles not in English; and studies unrelated to interprofessional collaboration (focused solely on pharmacists or GPs individually).

### 2.3. Study Selection Process

**Figure 1:**
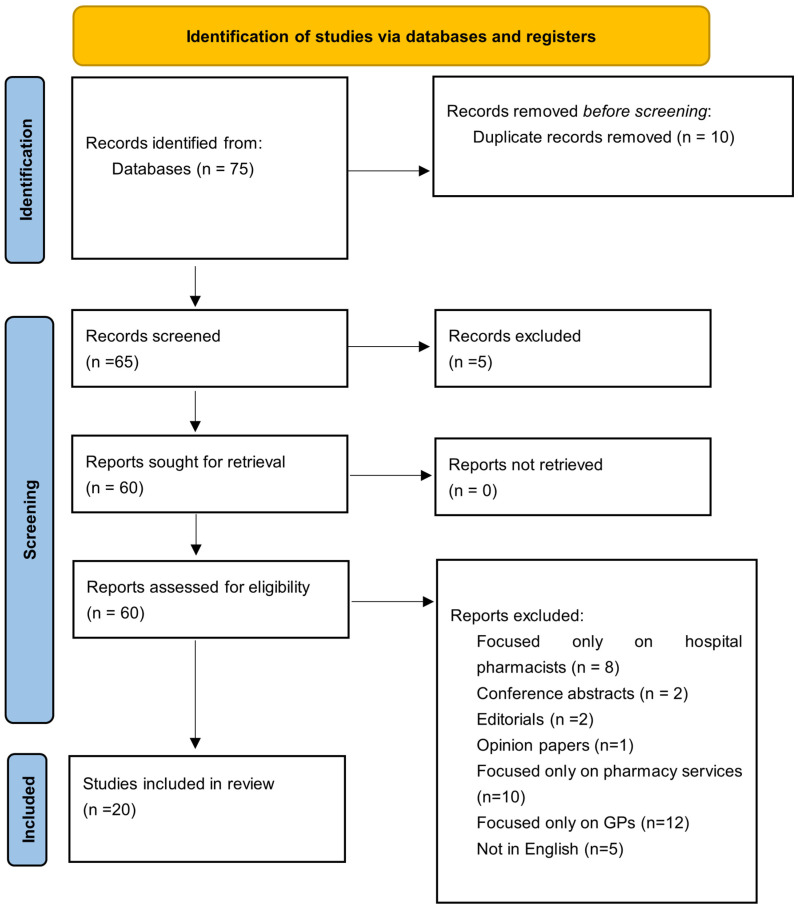
PRISMA ScR flow chart.

All identified articles were screened by two of the authors, and duplicates were removed. The remaining records were screened in two stages. The first stage involved screening titles and abstracts to exclude irrelevant studies. This was followed by a full-text review to determine final eligibility in accordance with the inclusion criteria. Disagreements were resolved through discussion and consensus, with consultation of a third reviewer when necessary. The selection process was documented in a PRISMA-ScR flow diagram, which outlines the number of studies identified, screened, excluded, and included (Figure 1).

### 2.4. Study Synthesis

The data were processed through a descriptive and thematic synthesis. First, we extracted relevant details and placed them in structured tables that capture each study’s characteristics and main results. Information related to pharmacist–general practitioner collaboration was then grouped and contrasted across studies to spot recurring patterns. Using an inductive approach, we conducted a thematic analysis, organizing the findings into broader themes that reflect both effective practices and challenges of collaboration. The results are presented narratively, illustrated by a table and a figure that summarize the key points. Consistent with the exploratory nature of a scoping review, we did not perform any quantitative synthesis or meta-analysis.

### 2.5. Critical Appraisal

In line with scoping review methodology and PRISMA-ScR recommendations, a formal risk-of-bias assessment was not conducted. This decision was based on the exploratory aim of the review and the heterogeneity of included study designs. The implications of this methodological choice are addressed in the Limitations section. The PRISMA-ScR checklist is provided as Supplementary Material.

## 3. Results

A total of 20 studies met the inclusion criteria and were analyzed. The findings were organized into two major thematic categories: (1) Good Practices and (2) Challenges in pharmacist–GP collaboration. Each category includes several subthemes, as identified through thematic synthesis (Table 1).

### 3.1. Good Practices in Pharmacist–GP Collaboration

Across the studies reviewed, pharmacist–general practitioner (GP) collaboration has been reported to contribute to patient care, particularly in medication safety, deprescribing, and chronic disease management. Embedding pharmacists within general practices [19,20,21] or involving them through structured models (e.g., BESTOPH-MG, MPC, POINT) contributed to medication reconciliation, early detection of cognitive impairment, and reduced medication-related hospitalizations. Interprofessional collaboration also led to higher patient satisfaction and trust, with patients in studies from Australia, Sweden, and Spain expressing appreciation for pharmacists’ input, particularly when GPs and pharmacists worked as a cohesive team [22,23,24]. A systematic review of randomized controlled trials compared standard depression care with a collaborative model that incorporates clinical pharmacists into general-practice teams, aimed to improve outcomes for treatment-resistant and major depressive disorder in Central Europe. The authors found that the pharmacist-inclusive approach consistently reduced depressive symptoms and offered a promising framework for expanding multidisciplinary care in primary-care settings [25]. A cross-sectional study in England evaluated the integration of general practice pharmacists, showing that their clinical role—medication reviews, medication reconciliation, and telephone support—has clearly expanded and is supported by evidence-based practice and by the presence of an experienced pharmacist mentor [26].

Positioning pharmacists in general practice clinics has been identified as an enabler of collaboration, resulting in informal interpersonal communication, mutual decision-making, and the formation of trusting working environments [24,27]. A study from Sloeserwij et al. (2021) [25] indicated that full integration of non-dispensing pharmacists into primary care teams facilitates effective collaboration with general practitioners. Non-dispensing pharmacists were embedded within practices and undertook a range of activities, including conducting clinical medication reviews, providing patient consultations, delivering staff education, and leading practice-level quality improvement initiatives tailored to local needs. Close, face-to-face collaboration enabled pharmacists to communicate recommendations directly to GPs, who implemented them more frequently than those from community pharmacists.

It was critical to clearly separate organizational and responsibility roles between pharmacists and GPs [25,28]. GPs were more collaborative when pharmacists had defined clinical roles, such as medication review or chronic disease management [29]. Interprofessional understanding and teamwork were assisted by regular meetings, case discussions, and joint care planning [28,30]. In countries such as Canada, Australia, and the UK, governments funded initiatives and developed policies that supported pharmacists in primary care, leading to more sustainable collaborations [30,31,32]. Studies from other countries examined collaborations for a particular patient group or over a shorter period [33,34,35].

Ramos et al. (2021) implemented an interprofessional collaboration (IPC) protocol involving community pharmacists, GPs, and neurologists across community pharmacies in Spain to screen for cognitive impairment [24]. IPC increased both referrals to neurologists and confirmed diagnoses compared with non-IPC sites, indicating that organized collaborations facilitate early detection of cognitive decline. Hazen et al. (2021) evaluated the “POINT” integrated care model in the Netherlands, which involved clinical pharmacists within GP practices and was delivered through a 15-month training program [25]. The intervention led to a 32% reduction in hospitalizations attributable to medication-related problems, facilitated quality improvement projects and medication reconciliation, and enhanced professional identity and collaboration with GPs.

Numerous studies have emphasized the critical role of effective communication and interprofessional collaboration, reporting that collaborative practices are directly associated with improved patient outcomes and increased satisfaction [29,30]. Results reported by Sloeserwij et al. (2021) indicate that pharmacist-integrated teams achieved statistically significant improvements in prescribing quality relative to control teams [25]. Common improvements in comparable studies include reduced use of high-risk medications, better alignment with clinical guidelines, and lower overall prescribing errors [29,30,31,32,33]. The study therefore supports the role of non-dispensing pharmacists as a valuable component of primary-care teams, contributing to safer and more evidence-based medication use.

Singapore private GPs are reported to view community pharmacists as able to improve primary care through medication reconciliation, device counseling, and coordinated drug purchasing. Still, collaboration is constrained by concerns about competitive pressures, low patient acceptance, the absence of shared electronic health records, and insufficient funding [21].

A cross-sectional survey of French general practitioners examined attitudes toward interprofessional collaboration in managing patients with multimorbidity and polypharmacy and identified distinct collaboration profiles with other health professionals, including pharmacists [31]. Facilitating factors for pharmacist–GP collaboration included positive GP attitudes toward interprofessional cooperation, particularly among those who recognized pharmacists’ expertise in medication management and actively communicated with them regarding prescriptions.

Research in China shows that integrating hospital pharmacists can contribute to medication safety and chronic-disease management in primary care; however, success depends on addressing skill gaps, fostering interprofessional trust, and providing supportive infrastructure. Community pharmacists recognize the importance of pharmaceutical-care services but are constrained by systemic and resource-related barriers [5]. Enhancing clinical training, integrating electronic health record access, and establishing reimbursement mechanisms are essential steps to enable pharmacists to shift from a dispensing-focused role to a more patient-centered, clinical practice model. This transition could support medication safety, chronic-disease management, and overall health outcomes within the Saudi primary-care landscape [30]. Structured collaboration models, such as Belgium’s MPC and France’s BESTOPH MG trial, demonstrate that introducing teamwork gradually, with appropriate support, helps build trust and strengthen cooperation among healthcare professionals [19]. From the patient’s perspective, there is a clear desire for a triangular conversation among GPs, community pharmacists, and patients to ensure consistent and coordinated advice, particularly regarding antibiotics and deprescribing [18].

The interview-based study of 16 New Zealand GPs demonstrates that pharmacists are regarded as complementary experts, patient-proximate partners, and a critical “safety net” that underpins good prescribing practice [32].

Although the clinical role of pharmacists is widely appreciated, real-world collaboration often does not fully match these positive attitudes. This gap highlights the need for stronger system-level support and clearer policies that encourage truly integrated care. Together, these studies underscore the value of GP–pharmacist partnerships and suggest practical steps to enhance the sustainability of collaboration across healthcare settings.

### 3.2. Challenges in GP–Pharmacists’ Collaboration

An overlap in perceptions of clinical responsibilities was reported to increase pressure among medical professionals. Several studies report that physicians perceive pharmacists as encroaching on their decision-making authority. Bergsholm et al. (2023) [18] applied position theory to examine differing perceptions among community pharmacists (CPs), general practitioners (GPs), and patients regarding pharmacists’ roles in antibiotic use. GPs are reluctant to involve pharmacists in clinical decisions.

The organizational challenges discussed by researchers are related to the unclear and cumbersome administrative procedures involved in implementing collaborative practices [18].

The lack of shared electronic health records or direct communication channels is a barrier to coordinated care [36,37,38]. Scarce or short-term funding for pharmacy services, especially in low- or middle-income countries, limits the sustainability and scalability of projects [36].

Not all pharmacists possessed the necessary clinical training; several studies reported that pharmacists lacked confidence and competence to assume clinical roles, particularly in diagnosis and counseling [14,15,16,17,18,19,20,21,22,23,24,25,26,27,28,29,30,31,32,33].

An umbrella study by Swiss researchers reported that collaboration is hindered by limited time, insufficient interprofessional training, unclear role boundaries, and poor communication, yet is facilitated by co-location, joint training, and mutual respect for professional expertise [14].

A qualitative interview study examined how hospital pharmacists were integrated into three Shenzhen community health centers and explored the perceptions of pharmacists, general practitioners, and health administrators regarding this model [33]. The barriers identified were pharmacists’ inadequate competence in primary care tasks, low patient recognition of pharmacists’ role, resistance from GPs, and insufficient technological support for information sharing [33].

**Figure 2:**
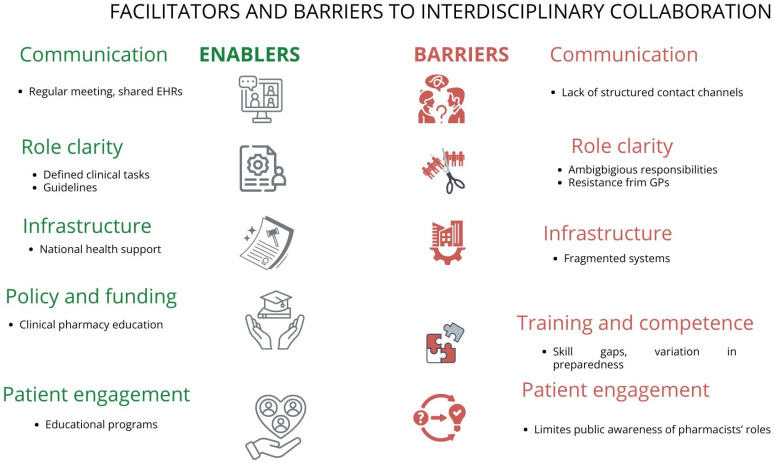
Facilitators and barriers to interdisciplinary collaboration of pharmacists and general practitioners in primary care.

Patients’ perceptions were studied. In some cases, patients do not trust pharmacists in non-dispensing roles. While the integration of pharmacists into general practice teams is relatively nascent in Australia, the established models and identified challenges provide a foundation for developing more robust interprofessional collaborations [20]. This integration is crucial given the positive effects of collaborative care on patient outcomes, particularly in rural and remote communities where healthcare access is limited and health disparities are pronounced [34]. While GPs are generally open to collaborating with pharmacists, the actual frequency of collaboration often lags. Success hinges on institutional support (e.g., communication protocols, joint education), formalized structures (e.g., MPC, embedded pharmacist roles), and sustained interaction to foster trust and integrate pharmacists (Figure 2) [14,15,16,17,18,19,20,21,22,23,24,25,26,27,28,29,30,31,32,33,34].

## 4. Discussion

### 4.1. Summary of Findings

The present scoping review mapped current evidence on interprofessional collaboration between pharmacists and general practitioners (GPs), identifying both persistent challenges and context-dependent opportunities for improvement. Consistent with international trends, pharmacist–GP collaboration appears to be increasingly reported across diverse healthcare systems, although its implementation remains uneven and strongly shaped by structural, cultural, and professional factors [39,40,41]. This observation aligns with earlier reviews suggesting that collaborative models are expanding globally yet remain highly sensitive to local system design and regulatory context [42,43].

Our findings confirm previous research demonstrating that pharmacist-led medication reviews, particularly for older adults and patients with polypharmacy, are a commonly reported collaborative activity in primary care [6]. However, as noted in earlier studies, the extent to which these services are integrated into routine practice depends largely on GP engagement and endorsement. Hughes et al. (2023) [17] similarly reported that unclear role expectations, limited feedback mechanisms, and concerns regarding professional boundaries may constrain sustained collaboration, even where clinical benefits are recognized. Rather than indicating resistance per se, such findings suggest that collaboration requires ongoing negotiation of roles and responsibilities.

Geographical proximity and shared infrastructure emerged as recurring enablers of collaboration in this review, findings consistent with prior studies examining co-located or practice-embedded pharmacists [19,20,21]. Earlier research has shown that physical proximity facilitates informal communication, relationship-building, and mutual trust, which, in turn, may support more timely clinical input from pharmacists [44]. Similarly, access to shared electronic health records has been widely cited as facilitating collaborative practice, enabling pharmacists to provide medication-related recommendations with greater clinical context and reduced duplication of effort. These observations are consistent with findings from integrated care models in countries with more mature digital health infrastructures [45].

Role clarity was another prominent theme identified in this review and has been repeatedly highlighted in the literature as a determinant of successful interprofessional collaboration. Studies conducted in jurisdictions with explicit scopes of practice, reimbursement mechanisms, and formalized collaborative agreements—such as the UK, Canada, and Australia—report higher levels of GP acceptance and more explicit task allocation [20]. However, as previously reported, the expansion of pharmacists’ clinical roles may also generate uncertainty or perceived professional overlaps, particularly in systems without clear governance frameworks. Several studies included in this review echo earlier findings that some GPs express concern about role encroachment, which may manifest as reluctance to delegate clinical responsibilities or engage fully in collaborative models.

In line with earlier international research, insufficient and unsustainable funding was identified as a significant barrier to long-term collaboration [36,37,38]. Short-term pilot programs, while valuable for demonstrating feasibility, often lack continuity and may limit the scalability of pharmacist services. Prior evaluations similarly note that the absence of stable reimbursement mechanisms can undermine professional commitment and organizational support, even when clinicians view collaborative interventions positively.

Educational and professional preparedness also featured prominently among reported barriers. Consistent with previous studies [18], variability in pharmacy curricula and clinical training across countries appears to influence pharmacists’ confidence and readiness to participate in patient-centered, team-based care. In settings where pharmacy education remains product-focused rather than clinically oriented, pharmacists may be perceived—and may perceive themselves—as less equipped for collaborative practice. Additionally, patient-level factors, including limited awareness of pharmacists’ clinical roles, have been reported elsewhere as influencing acceptance of expanded pharmacy services and were similarly reflected in the studies reviewed.

Overall, while the findings of this scoping review are broadly consistent with the existing literature highlighting the potential value of pharmacist–GP collaboration [43], they underscore that implementation is shaped less by individual professional willingness and more by system-level conditions, including funding models, regulatory clarity, education, and health information infrastructure [44]. By synthesizing evidence across multiple contexts, this review contributes to an understanding of why collaborative models succeed in some settings while remaining difficult to sustain in others.

### 4.2. Limitations

This scoping review is subject to several limitations that should be considered when interpreting its findings. First, the literature search was limited to English-language publications, which may have excluded pertinent studies published in other languages. Although multiple major databases were consulted, gray literature and unpublished works were not systematically retrieved, potentially compromising the comprehensiveness of the evidence base.

Second, in line with scoping-review methodology and the PRISMA-ScR guidelines, a formal risk-of-bias or quality appraisal of the included studies was not performed. Consequently, the review cannot provide judgments about the effectiveness or relative strength of specific collaborative interventions; its conclusions are descriptive rather than evaluative.

Third, the included studies exhibited considerable heterogeneity in study design, healthcare settings, national health systems, and outcome measures. This diversity precluded quantitative synthesis and limited direct comparability across studies. Moreover, many investigations reported only short-term or context-specific results, restricting the ability to infer the long-term sustainability of pharmacist–general practitioner collaboration.

Finally, variability in how collaborative models and outcomes were reported may have influenced the thematic synthesis.

Despite these constraints, the scoping review offers a comprehensive overview of the existing literature, delineates key enablers and barriers, and highlights critical gaps that should guide future research and policy development.

## 5. Conclusions

Modern, integrated primary care is supported by collaboration between pharmacists and general practitioners. While significant progress has been made in implementing pharmacist–GP partnerships, many barriers still hinder their full integration.

Good practices such as co-location, shared access to health records, precise role definitions, structured communication, and supportive policy environments enhance collaboration. However, professional role ambiguity, inadequate funding, communication problems, and insufficient training continue to challenge the sustainability of such models.

To contribute to the impact of pharmacist–GP collaboration, healthcare systems must adopt a comprehensive approach that includes policy reform, investment in interprofessional infrastructure, ongoing training, and public awareness initiatives. Supporting these collaborations has the potential to support medication management, reduce healthcare costs, and enhance patient outcomes across diverse primary care settings.

## 6. Future Directions

Future research should examine the economic impact of integrating pharmacists into primary care settings. In addition, opportunities to establish sustainable collaborative models grounded in mutual trust and multidisciplinary cooperation between pharmacists and general practitioners should be explored, taking into account the specific characteristics of different healthcare systems. Introducing collaborative care models early in professional education may also help address the challenges associated with interprofessional practice.

## Figures and Tables

**Table 1:** Characteristics of the included studies.

No.	Authors (Year)	Country	Study Design	Focus/Settings	Main Findings Related to Collaboration
1	Hasan Ibrahim et al. (2023) [17]	UK	Qualitative interviews	Practice-based pharmacists	Trust and role clarity enable integration.
2	Cai et al. (2023) [16]	China	Cross-sectional study	Polypharmacy management	Positive GP attitudes: Collaboration frequency is limited
3	Bergsholm et al. (2023) [18]	Norway	Cross-sectional survey	Antibiotic prescription	GPs identify that pharmacists lack clinical experience.
4	Damiaens et al. (2021) [19]	Belgium	Mixed methods	Collaborative care models	Local organizational support was insufficient for sustainability.
5	Sudeshika et al. (2022) [20]	UK, Canada, Australia	Cross-sectional survey	Medication reviews	Moderate integration: Communication improves effectiveness
6	Percival et al. (2023) [21]	Australia	Interviews	Pharmacist co-location	Improved management of medication-related problems
7	Chong et al. (2023) [22]	Singapore	Qualitative study	Community pharmacists and GPs collaboration	Significant concerns about GPs-CPs’ clinical collaborations
8	Carlqvist et al. (2024) [23]	Sweden	Qualitative study	Dementia care	Improved patient safety
9	Ramos et al. (2021) [24]	Spain	Comparative Case Study	Cognitive Impairment Screening	Increased detection through interprofessional collaboration
10	Rawlinson et al. (2021) [14]	Switzerland	Review	Interprofessional collaboration	Limited time, role overlap, and poor communication
11	Hazen et al. (2021) [25]	Netherlands	Observational	Training program for pharmacists	Reduced hospitalizations due to medication-related problems
12	Huon et al. (2023) [26]	Canada	Cluster randomized controlled trial	Deprescribing	Structured GP–pharmacist interventions feasible
13	Hassan et al. (2024) [27]	Ireland	Cross-sectional survey	Practice-based pharmacists	Positive perception of expanded pharmacists’ roles
14	Alshehri et al. (2021) [28]	UK	Cross-sectional survey	GPs perceptions	Positive acceptance of pharmacists in general practice
15	Stuhec and Zelko (2021) [29]	Slovenia	Systematic review	Depression treatment	Effectiveness in the pharmacotherapy management of patients
16	Moecker et al. (2022) [30]	Germany	Cross-sectional postal survey	Pharmacist-led medication management program	Positive collaboration
17	Carrier et al. (2022) [31]	France	Cross-sectional survey	Polypharmacy management	Variable GP attitudes towards cooperation
18	Addison and Taylor (2023) [32]	New Zealand	Semi-structured interviews	GP–pharmacist relationships	Pharmacists improve patient safety.
19	Deng et al. (2025) [33]	China	Qualitative	Hospital pharmacists in primary care	Barriers such as a lack of patient recognition and practice skills
20	Sloeserwij et al. (2020) [34]	Netherlands	non-randomized, controlled intervention study,	Influence on the quality of prescribing	consistent improvement

## Data Availability

No new data were created or analyzed in this study. Data sharing is not applicable to this article.

## Supplementary Materials

The following supporting information can be downloaded at: https://www.mdpi.com/article/10.3390/pharmacy14010024/s1, File S1: Preferred Reporting Items for Systematic reviews and Meta-Analyses extension for Scoping Reviews (PRISMA-ScR) Checklist.

## Abbreviations

The following abbreviations are used in this manuscript:

GP	General Practitioner
GPs	General Practitioners
CP	Community Pharmacist
PRISMA-ScR	Preferred Reporting Items for Systematic Reviews and Meta-Analyses Extension for Scoping Reviews
UK	United Kingdom
IPC	Interprofessional collaboration
MPC	Medication Process Collaboration
BESTOPH-MG	Benzodiazepine Stop in Older People in Primary Care–General Medicine
POINT	Pharmacotherapy Optimisation through Integration of a Non-dispensing Pharmacist
EHR	Electronic Health Record
